# What Do We Know about Peripartum Cardiomyopathy? Yesterday, Today, Tomorrow

**DOI:** 10.3390/ijms251910559

**Published:** 2024-09-30

**Authors:** Ratko Lasica, Milika Asanin, Jovanka Vukmirovic, Lidija Maslac, Lidija Savic, Marija Zdravkovic, Dejan Simeunovic, Marija Polovina, Aleksandra Milosevic, Dragan Matic, Stefan Juricic, Milica Jankovic, Milan Marinkovic, Lazar Djukanovic

**Affiliations:** 1 Department of Cardiology, Emergency Center, University Clinical Center of Serbia, 11000 Belgrade, Serbia; drlasica@gmail.com; 2 Faculty of Medicine, University of Belgrade, 11000 Belgrade, Serbia; masanin2013@gmail.com (M.A.); lidijasavic2007@gmail.com (L.S.); sekcija.kardioloska@gmail.com (M.Z.); dejan.simeunovic@med.bg.ac.rs (D.S.); maki.marijapolovina@gmail.com (M.P.); milosevic.aleksandra506@gmail.com (A.M.); dragan4m@gmail.com (D.M.); marinkovicm86@gmail.com (M.M.); 3 Department of Cardiology, University Clinical Center of Serbia, 11000 Belgrade, Serbia; lidijamaslac98@gmail.com (L.M.); stefan.juricic@gmail.com (S.J.); jankovic.s.milica@gmail.com (M.J.); 4 Faculty of Organizational Sciences, University of Belgrade, 11000 Belgrade, Serbia; jovanka.vukmirovic@fon.bg.ac.rs; 5 Clinical Center Bezanijska Kosa, 11000 Belgrade, Serbia

**Keywords:** peripartum cardiomyopathy, heart failure, pregnancy, treatment, prognosis

## Abstract

Peripartum cardiomyopathy is a disease that occurs during or after pregnancy and leads to a significant decline in cardiac function in previously healthy women. Peripartum cardiomyopathy has a varying prevalence among women depending on the part of the world where they live, but it is associated with a significant mortality and morbidity in this population. Therefore, timely diagnosis, treatment, and monitoring of this disease from its onset are of utmost importance. Although many risk factors are associated with the occurrence of peripartum cardiomyopathy, such as conditions of life, age of the woman, nutrient deficiencies, or multiple pregnancies, the exact cause of its onset remains unknown. Advances in research on the genetic associations with cardiomyopathies have provided a wealth of data indicating a possible association with peripartum cardiomyopathy, but due to numerous mutations and data inconsistencies, the exact connection remains unclear. Significant insights into the pathophysiological mechanisms underlying peripartum cardiomyopathy have been provided by the theory of an abnormal 16-kDa prolactin, which may be generated in an oxidative stress environment and lead to vascular and consequently myocardial damage. Recent studies supporting this disease mechanism also include research on the efficacy of bromocriptine (a prolactin synthesis inhibitor) in restoring cardiac function in affected patients. Despite significant progress in the research of this disease, there are still insufficient data on the safety of use of certain drugs treating heart failure during pregnancy and breastfeeding. Considering the metabolic changes that occur in different stages of pregnancy and the postpartum period, determining the correct dosing regimen of medications is of utmost importance not only for better treatment and survival of mothers but also for reducing the risk of toxic effects on the fetus.

## 1. Materials and Methods

To write this paper, PubMed, Web of Science, Scopus, and Google Scholar databases were searched in order to obtain data on peripartum cardiomyopathy (PPCM). The authors searched the listed databases using the given keywords: peripartum cardiomyopathy, heart failure, pregnancy, treatment, and prognosis. We focused on recent works and those with a higher degree of importance. We also included European and American guidelines regarding the treatment of heart failure and consensus for the treatment of PPCM. Randomized controlled trials, meta-analyses, registries, reviews, and studies with larger sample sizes were preferred, but in the absence of these for certain areas, we used available case series and case reports that might be relevant. A total of 2757 publications were identified. After removing duplicates and excluding studies based on their abstracts or through examination of their full text, 230 publications were eligible for inclusion in this review paper (Supplementary Figure S1).

## 2. Definition and Concept of Peripartum Cardiomyopathy

Although PPCM has not yet been defined as a distinct entity, a case of sudden pulmonary congestion was described as early as 1849 in a young woman 3 weeks postpartum [1]. Later studies also described cases of sudden idiopathic heart failure (HF) in patients during pregnancy and after childbirth [2,3]. Data by Hull and colleagues in 1937 provided clearer clinical and pathological descriptions of disease correlated with pregnancy and the postpartum period in previously healthy young women. Since then, this condition has been referred to in most studies as “postpartum heart disease” [4]. Although peripartum heart failure was mentioned in a paper by Seftel H. and colleagues in 1961 [5], the official term PPCM was defined in 1971 by J. G. Demakis and colleagues, who suggested that the term PPCM was more appropriate since some patients develop the disease in the final months of pregnancy, replacing the previously used term postpartum cardiomyopathy [6]. The Heart Failure Association of the European Society of Cardiology (ESC) defines PPCM as idiopathic cardiomyopathy accompanied by HF with a reduced left ventricular ejection fraction (LVEF) < 45% that occurs at the end of pregnancy or in the months after childbirth, miscarriage or abortion [7]. This definition does not set clear time intervals for the onset of cardiomyopathy, unlike the definition of the National Heart, Lung and Blood Institute and the Office of Rare Diseases, according to which idiopathic cardiomyopathy occurs in the last month of pregnancy or 5 weeks after delivery. Most PPCM patients have an LVEF < 45%, and cases with an LVEF in the range of 45–50% are rarely mentioned. [8]. The literature also mentions a case of PPCM with preserved LVEF, which does not fit into the previously established PPCM definition [9]. The study results by Elkayam U. and colleagues, which compared the characteristics of idiopathic dilated cardiomyopathy (DCM) and classically defined PPCM, confirmed that there is no significant clinical deviation between these two groups of patients [10].

## 3. Epidemiology of PPCM

Epidemiological data related to PPCM are largely inconsistent and vary both between different countries and among different ethnic groups. Additionally, the diversity of inclusion criteria in various epidemiological studies has contributed to a wide range of differing results. According to data from a large cross-sectional study, the prevalence of heart diseases among pregnant women was 1.33 per 1000 deliveries, compared to all causes of hospitalizations among pregnant women. It is important to note that the hospitalization rate for all forms of cardiomyopathies among pregnant women was 0.46 per 1000 deliveries. Of patients hospitalized for some form of cardiomyopathy, 0.18 were due to PPCM [11]. The prevalence of PPCM is not low either, and in the United States is estimated to be around 1 in 2000 deliveries [12]. In the United States, the incidence of PPCM is highest among African Americans (1 in 439–1421 deliveries) [13]. Women of African descent have a 15.7 times higher relative risk of PPCM compared to women of other ethnic backgrounds [14]. The highest incidence was reported in studies conducted in Nigeria and Haiti, where the estimated incidence of PPCM was 1:100 and 1:299, respectively [15,16]. In Europe, a Danish population study showed an incidence of PPCM of 1:10,149 deliveries [17]. The lowest incidence of PPCM was reported in Japan (1:20,000 deliveries) [18]. In the Scottish PPCM follow-up study (follow-up period 20 years), the incidence of PPCM was approximately 1 in 4950 cases (95% CI 1.76–2.29) [19]. Data from Britain are consistent with the previously mentioned data from the Scottish study (2.12 per 10,000 deliveries, 95% CI 2.03–2.21) [19].

## 4. Risk Factors for Developing PPCM

It is believed that numerous factors contribute to the occurrence of PPCM, but the exact cause of this condition remains unknown. Current significant risk factors for PPCM include being of Black race, preeclampsia, high blood pressure, multiple pregnancies, older maternal age, a prior history of malignancy, socioeconomic status, etc. [10,20]. According to the study by O. C. Irizarry and colleagues, PPCM occurred at an earlier age in pregnant women of African descent compared to Caucasian women (average age 27.6 vs. 31.7 years, *p* < 0.01, respectively). Additionally, it is noteworthy that African American women more frequently had a greater degree of left ventricular impairment, as evidenced by echocardiography (LVEF < 30%) [21].

Numerous studies have shown the correlation of hypertension and preeclampsia with the occurrence of PPCM [8,20,22,23,24,25,26]. According to a study by Melamed N. and colleagues, 17–46% of women who have elevated values of arterial blood pressure during pregnancy develop preeclampsia, while preeclampsia is less common in normotensive patients (5–8%) [27]. The risk of PPCM is up to four times higher in women with preeclampsia compared to women whose pregnancy is not complicated by this disease. Data also indicate a similar variation in the incidence of preeclampsia and PPCM across different regions [28]. In a meta-analysis by Bello I. and colleagues, it was shown that the prevalence of preeclampsia in women with PPCM is around 22%, compared to the global average rate of 5% [22]. Other studies have provided similar data on the frequency of preeclampsia in patients with confirmed PPCM [29]. Across the United States, 7–14.5% of women with PPCM have reported multiple pregnancies [30]. A study by Jackson AM and colleagues, which examined 255 women with PPCM, showed through multivariate analysis that excess body weight, high blood pressure, multiple pregnancies, and preeclampsia are all independently linked to an increased risk of developing PPCM [19]. The study also pointed to the fact that PPCM patients more frequently had multiple pregnancies (8% vs. 2%) [19].

The results of a meta-analysis by Putra ICS and colleagues indicate that excess body weight in women before pregnancy is significantly associated with the development of PPCM compared with non-obese women [31]. The association of diabetes mellitus, anemia, and deficiencies of certain minerals like selenium with the occurrence of PPCM has been demonstrated in some studies [32,33,34,35]. On the other hand, some studies do not link selenium deficiency with the occurrence of PPCM. Such results were shown by a study conducted on the population of Haiti [36]. Advanced maternal age is considered a risk factor for the development of PPCM [37,38]. The study by Pfeffer TJ and colleagues reported that patients with PPCM have a higher prevalence of malignancies compared to women of the same age without PPCM [39]. According to the study, the association between PPCM and a high incidence of malignancies could be attributed to genetic mutations that are associated not only with cancer but also with the development of both DCM and hypertrophic cardiomyopathy [39]. A study examining the incidence of peripartum cardiac dysfunction in patients who had some form of malignancy in early childhood, adolescence, or young adulthood showed a significantly higher relative risk of developing cardiac dysfunction in the peripartum period compared to the general population [40]. This association could also be due to the use of various cardiotoxic drugs. In this regard, the Children’s Oncology Group recommends follow-up by a cardiologist for women who have previously been exposed to significant doses of doxorubicin or who have been treated with radiotherapy. Based on current knowledge, it is reasonable to take anamnestic data on the cancer history of every pregnant woman and perform cancer screening in PPCM patients. According to study data, PPCM patients have a lower level of socioeconomic well-being compared to controls [41]. It has also been shown that patients with poorer socioeconomic status have worse outcomes in the treatment of PPCM [42]. To enable early diagnosis of PPCM, Davis MB and colleagues proposed a validated risk prediction model that identifies women at increased risk for PPCM at the time of delivery [43]. These patients should be monitored for weight gain, mood disorders, risk factors for diabetes mellitus, and socioeconomic status.

## 5. Pathogenetic Mechanisms of PPCM Development

**Figure 1:**
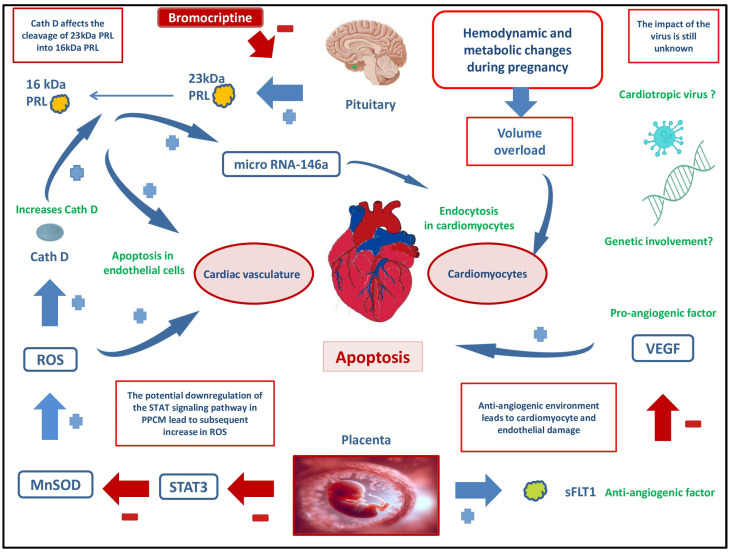
Potential pathogenetic mechanisms in the development of peripartum cardiomyopathy. **Legend**: **STAT**—Signal Transducer and Activator of Transcription; **MnSOD**—Manganese SuperOxide Dismutase; **ROS**—Reactive Oxygen Species; **microRNA**—single-strand endogenous and non-coding RNA molecules; **sFLT1**—soluble Fms-Like Tyrosine kinase 1; **VEGF**—Vascular Endothelial Growth Factor; **PPCM**—PeriPartum CardioMyopathy; **PRL**—Prolactin; **Cath D**—Cathepsin D; **−** decrease; and **+** increase; The figure shows potential mechanisms that may be involved in the development of PPCM. Potential downregulation of STAT3 signaling pathways leads to a decrease in MnSOD synthesis leading to an increase in ROS. ROS can increase the synthesis of Cath D which further plays a role in the cleavage of prolactin 23 kDa to the highly reactive form 16 kDa PRL. This form of PRL can cause direct damage to the blood vessel endothelium, and potentiating the increase in microRNa-146 leads to cardiomyocyte apoptosis. As part of the hemodynamic changes during pregnancy, the heart muscle is loaded with volume, which is accompanied by hypertrophy and increased oxygen needs. Increased expression of sFLT1 (a known anti-angiogenic factor) in pregnancy leads to a decrease in VEGF, which definitely reduces the angiogenesis of volume-loaded cardiomyocytes. This anti-angiogenic environment may lead to additional cardiomyocyte apoptosis. Cardiotropic viruses can also be responsible for the development of PPCM, although their role in the development of this disease has not been fully investigated.

Possible pathogenetic mechanisms leading to PPCM include genetic predisposition, hormonal imbalance, lack of some microelements, infections, diseases of autoimmune origin, stress, etc. [44,45,46]. PPCM often presents as a poor response to hemodynamic stress during pregnancy. Significant changes in physiological functioning occur during pregnancy and postpartum. During pregnancy, substantial hemodynamic changes significantly increase the load on the heart (Figure 1). Not infrequently, polyglobulia, as well as increased cardiac output and heart mass occur. Stroke volume and heart rate both elevate by around 15% to 30% [47].

Plasma volume during pregnancy increases by up to 48%, which can lead to volume loading of the left ventricle [48]. The increased load on the heart during pregnancy results in an increase in heart size and mass and an enlargement of the left ventricle. This is a form of physiological hypertrophy considered reversible. A characteristic of this form of hypertrophy is the absence of fibrosis, decreased cardiac function, and reduced angiogenesis, which are seen in pathological cardiac hypertrophy [47,49]. In pathological hypertrophy of the heart muscle, there is an increased production of atrial natriuretic factor, alpha and beta-myosin heavy chains, and other precursors, while in physiological hypertrophy such changes are not shown [49]. During pregnancy, the metabolism of sugars, fats, and ketone bodies is often disrupted. Insulin resistance in pregnant women usually begins early in pregnancy, which can endanger the fetus [50]. Serum lipids dramatically increase during pregnancy. Triglycerides are elevated by two to three times, total cholesterol by 25–50%, and LDL by 30–40% [51,52]. This inadequate lipid profile can significantly lead to endothelial damage. The reduction in total peripheral resistance (35–40%) along with increased vascular compliance prevents an increase in arterial pressure during pregnancy [47,48]. Therefore, endothelial cells increase the production and bioavailability of vasodilatory products such as nitric oxide [53]. In addition to uterine blood flow, splanchnic, renal, pulmonary, and skin blood flow also increase during pregnancy [47]. Most of the aforementioned hemodynamic changes that represent hemodynamic stress occur within the first trimester of pregnancy when cardiomyopathy should most commonly manifest. However, according to the definition, PPCM most often occurs in the third trimester of pregnancy and later after delivery, when hemodynamic stress is significantly lower. As a result, numerous hypotheses have emerged over time to explain the occurrence of PPCM during that time period. Therefore, apart from hemodynamic changes and the mentioned genetic factors, many other factors have been considered as causes of its development. Viral infections with cardiotropic viruses can cause inflammation of the heart muscle, which can be asymptomatic, have a mild to moderate clinical course, or manifest as a fulminant form with rapid onset of heart failure and often fatal outcomes. Myocarditis is a disease that often leads to reversible changes in the heart muscle, but not infrequently it can irreversibly lead to dilatation of the left ventricle resulting in DCM [54]. Regarding progression to DCM, data are heterogeneous, but some studies suggest that even 30% of patients with previous myocarditis develop post-inflammatory DCM [55]. According to a study by Berkhard D. and colleagues, which examined endomyocardial biopsy (EMB) samples from 26 patients with PPCM, the presence of viral genomes was shown using the PCR method (in 30.7% of patients) [56]. The IPAC study detected a finding suggestive of myocarditis in only one patient [57]. In a study by Cavanaugh H and colleagues, it was found that patients with PPCM showed borderline myocarditis on EMB according to the Dallas criteria, and evidence of myocarditis was also demonstrated by magnetic resonance imaging [58]. In a study that included 34 women with PPCM, late accumulations of gadolinium in the heart muscle were registered in 70%, and the presence of myocardial edema in 25% of patients [59]. However, there are also studies in PPCM patients in whom no signs of myocarditis (late gadolinium accumulation and myocardial edema) were registered by magnetic resonance imaging [60]. This discrepancy in findings could most likely be explained by different timing of MRI in relation to the onset of disease symptoms [61]. Cardiac magnetic resonance imaging is a significant diagnostic tool for identifying the etiology of diseases, especially when the clinical picture may correspond to post-myocarditis PPCM [62].

The G protein Gq links intracellular signaling pathways to several cell surface receptors involved in cardiac myocyte hypertrophy, including the α1-adrenergic receptor, angiotensin II type 1 receptor, and endothelin-1 receptor [63]. Research has revealed that mice with cardiac-specific overexpression of the Gq subunit, Gαq, display early signs of cardiac hypertrophy and impaired contractile function. Ventricular hypertrophy depends on the level of expression of the Gαq, and a fourfold increase in the Gαq leads to an increase in heart muscle mass [64]. In the case of increased expression of the mentioned and increased volume load, cardiac muscle hypertrophy always occurs. Although initially compensatory, hypertrophy can progress to heart failure, and in animal models it coincides with cardiomyocyte apoptosis [65]. According to this, excessive activation of Gq signaling pathways may be responsible for the onset of cardiomyocyte apoptosis and the subsequent progression of the disease. Examining the phenomenon of apoptosis, the role of signal transducer and activator of transcription (STAT) proteins was particularly investigated. Their role in protecting the heart muscle from various harmful effects is significant. STAT affects the proliferation of cardiomyocytes, their metabolism, and protection against oxidative stress. Their activity is largely regulated by various pro-inflammatory and anti-inflammatory cytokines, and they can be activated by factors such as IL-6, IL-11, IL-22, and others. In addition to affecting cardiomyocytes, STAT3 also affects the function of endothelial cells and fibroblasts in the heart. Regarding antiapoptotic effects, STAT3 can improve the regulation of genes responsible for the synthesis of antioxidant enzymes, thereby reducing the production of reactive oxidative radicals. STAT3 significantly reduces the synthesis of reactive oxygen species by inducing the enzyme manganese superoxide dismutase (MnSOD) (Figure 1) [63,66]. Its role in blood vessel angiogenesis and cardiac muscle hypertrophy has also been described [63,67]. In a study by Ersbøll A. S. and colleagues on a mouse model with inhibited STAT3 factor activity, PPCM developed, whereas the control group of mice did not develop PPCM. This study demonstrated a significant effect of STAT3 on the regulation of superoxide dismutase 2 gene expression. The absence of STAT3 led to an increase in reactive oxygen species and increased secretion of cathepsin D (Figure 1).

Unbalanced oxidative stress during or after pregnancy is associated with proteolytic cleavage of the hormone prolactin, which may represent a specific pathomechanism for the development of PPCM [68]. Prolactin is a proapoptotic and proinflammatory factor. Prolactin is found in a full-length 23-kDa form, which can be split by cathepsin D into a 16-kDa fragment. This fragment triggers apoptosis in endothelial cells (Figure 1) [68]. Additionally, studies have linked the 16-kDa prolactin fragment to its role in the expression of single-strand endogenous and non-coding RNA molecules (microRNA), especially microRNA146a. The increase of microRNA146a leads to increased endocytosis in cardiomyocytes and consequent apoptosis (Figure 1). The elevated levels of microRNA146a in PPCM significantly decrease with bromocriptine treatment [69,70,71]. Soluble fms-like tyrosine kinase 1 (sFlt1) is an anti-angiogenic factor secreted by the placenta and endothelial cells [72]. When sFlt1 levels were measured in healthy women between the fourth and sixth week postpartum, it was found that their levels decreased to normal values within a maximum of 3 days after delivery. In contrast, women who developed PPCM had sFlt1 levels that were significantly elevated compared to those without PPCM [73]. According to a study by Damp J and colleagues involving 98 women with PPCM, higher sFlt1 levels were found to correlate with the occurrence of major adverse events and more intense disease symptoms [74]. During late pregnancy, there is placental release of another factor, an inhibitor of vascular endothelial growth factor (VEGF) (Figure 1). This release is greatest in cases of multiple pregnancy and preeclampsia [75].

## 6. Genetic Mechanisms of PPCM Development

The genetic basis of PPCM is still largely unexplored. In the case of DCM, familial forms have been clearly demonstrated, as many studies have shown [76,77,78]. Familial association with the occurrence of PPCM among close relatives has also been described, primarily through various case reports [79,80,81,82,83,84]. There is an increased incidence of PPCM in women of African American descent, as well as in studies conducted in Nigeria and Haiti, which may further indicate a genetic basis, different socioeconomic conditions, or greater exposure to a predisposing factor in certain regions of the world. On the other hand, what does not support a genetic predisposition is the possibility that a person who develops PPCM during the first pregnancy may not develop it in subsequent pregnancies. According to studies, PPCM relapse in repeated pregnancies occurs in up to 30% of cases [85,86,87]. A recent meta-analysis by Wijayanto M. A. and colleagues demonstrated that there is a higher incidence of relapse and higher mortality in patients who had PPCM in a previous pregnancy [88]. Although PPCM is today considered a separate clinical phenomenon from DCM, there are certain overlaps between these entities. About 20% of women who develop PPCM during or after delivery have clinical signs and symptoms of HF and echocardiographic features of DCM. A positive family history of cardiomyopathies in first-degree relatives was observed in 16.5% of women with PPCM [89]. The most common mutations involved in the development of DCM occur in genes such as those encoding sarcomere proteins (genes encoding myosin proteins (***MYH6***, ***MYH7***, and ***MYBPC3***), actin proteins (***ACTC1*** and ***ACTC2***), troponin, and tropomyosin proteins (***TPM1***, ***TNNC1***, and ***TNNT2***)) and occur in 5–10% of cases [90]. According to the study by Spaendonck-Zwarts et al., in which cardiological screening was conducted on first-degree relatives of three patients with PPCM who did not fully recover heart function, DCM was discovered in all three families, which had not been previously diagnosed [91]. The study by Morales A. and colleagues examined the prevalence of known genetic mutations for DCM in patients with PPCM and pregnancy-associated cardiomyopathy (PACM). Among the largest genes in humans, the Titin gene (***TTN***) encodes the protein titin. Titin spans between the Z and M sarcomeres and functions in maintaining sarcomere length, which is closely related to muscle contraction strength. Its role also involves maintaining passive tension and elasticity to sustain diastolic and systolic functions. In DCM, shortened ***TTN*** mutations (***TTNtv***) are the most common, contributing to approximately 25% of familial cases of DCM [92]. ***TTNtv*** leads to an increase in myofibril elasticity [93]. In the research conducted by Ware JS and colleagues, it was demonstrated that the occurrence of rare truncating variants in PPCM patients (15%) is markedly higher compared to the reference population (4.7%). However, this percentage is comparable to that observed in the cohort of DCM patients (17%). Such a finding is significant as it highlights the association of rare truncating variants with both PPCM and DCM, raising concerns about the potential link between these disorders [94]. Of the 26 rare truncating variants diagnosed in eight genes among women with PPCM, 17 (65%) affected ***TTN***. An example of a frameshift mutation in ***TTN*** in a patient with PPCM has been described [95]. Van Spaendonck-Zvarts KI and colleagues indicate that genetic mutations are common in families with PPCM and DCM. They identified four pathogenic mutations in four of eighteen families (22%): three in ***TTN*** and one in Bcl2-associated athanogene 3 (***BAG3***) [96]. Mutations in genes encoding nuclear proteins lamin A and C, such as ***LMNA*** on chromosome 1, are involved in the development of DCM in 5.9–8% of patients [97,98]. Lamin A and lamin C are key structural proteins of the nuclear lamina. They form a network of intermediate filaments located on the inner surface of the nuclear membrane. Mutations in ***LMNA*** often cause severe and progressive DCM with a poor outcome. It is believed that this gene mutation is responsible for up to 33% of DCM cases with atrioventricular conduction disorders [99,100]. The study by Castrini AI and colleagues investigated the prevalence of cardiac dysfunction and arrhythmias in women with pathogenic or likely pathogenic variants of the genes encoding lamin A/C proteins (***LMNA+***). Within the studied cohort, no higher prevalence of adverse events was found. Generally, women with ***LMNA+*** tolerated pregnancy well, with a small proportion of patients experiencing arrhythmias [101]. The possible association of the ***LMNA*** mutation with a fatal outcome from PPCM has been described in a large familial study [102]. Mutations in ion channel genes, such as sodium channel (***SCN5A***) and phospholamban (***PLN***), occur in patients with DCM with a frequency of 1.7% and 1%, respectively [103,104]. Early onset of the disease is associated with frequent supraventricular and ventricular rhythm disorders and is associated with DCM accompanied by a mutation in the ***SCN5A*** gene. Age is a determining factor in the penetration of the **SCN5A** gene when DCM is associated with this mutation. It is important to note that with increasing age, the expression of the phenotype becomes more pronounced [90]. Phospholamban is a protein that serves as an inhibitor of the SERCA pump in the heart muscle and is encoded by the ***PLN*** gene. In a study investigating ***PLN*** gene mutations in 315 patients with DCM, hypertrophic cardiomyopathy, PPCM, and arrhythmogenic right ventricular cardiomyopathy, an association between this mutation and DCM was demonstrated [105]. Mutations in genes for cytoskeletal proteins (desmin (**DES**) gene; Cypher/ZASP (***LDB3***); and dystrophin (***DMD***)) have been described as causes of DCM. Desmin is a protein found in the extracellular skeleton of cardiac muscle, and most ***DES*** mutations are missense mutations within the central domain [106]. Earlier cases of PPCM have been described in patients who carry genetic mutations for DMD [107,108]. Additionally, cases of PPCM have been reported in patients who are carriers of the ***LAMP2 X-linked gene*** [109].

Besides these, there are other genetic mutations correlated with patients with PPCM in studies, such as *family molecular chaperone regulator 3* (***BAG3***) [90]. The occurrence of this ***BAG3*** gene mutation in patients with PPCM was proven in a study by van Spaendonck-Zwarts KY and colleagues [96]. According to studies, the C825T polymorphism in the ***GNB3*** gene is correlated with poorer outcomes in women with PPCM who carry this mutation [110,111,112]. A multicenter study by Goli and colleagues from 2021 retrospectively identified PPCM. According to this study, about 10.4% of women carried truncating mutations in the ***TTN*** gene, but the study also identified the presence of truncating variants in filamin C, desmoplakin, and ***BAG3*** [112]. It is difficult to determine the exact role of these mutations in the development of PPCM, considering that the evidence comes from individual cases and familial studies, and overlaps with DCM.

## 7. Treatment of PPCM

The management of patients who develop PPCM largely depends on the timing of its onset, whether it occurs during pregnancy or after delivery. There are many clinical and ethical dilemmas regarding the treatment of this disease, making a multidisciplinary approach necessary (involving gynecologists, cardiologists, anesthesiologists, cardiac surgeons, etc.) [113]. The treatment of PPCM is derived from a combination of expert opinions and recommendations for other forms of HF. Therefore, the approach to treating PPCM should follow the leading guidelines for the treatment of HF from the European Society of Cardiology and the American Heart Association for both the acute and chronic phases of the disease [7,30,114,115,116,117,118,119,120]. The treatment of HF during pregnancy requires special modifications to ensure fetal safety. After delivery, discontinuation of breastfeeding should be considered, but if the decision is to continue breastfeeding, it is important to note that most HF medications can be used during lactation.

### 7.1. Acute Heart Failure (AHF)

According to the 2021 ESC guidelines, AHF represents a rapid or gradual onset of symptoms and/or signs of HF severe enough for the patient to seek emergency medical assistance and necessitate hospitalization [114]. In patients with PPCM who develop pulmonary edema or cardiogenic shock, it is important to follow the leading guidelines for treating AHF [114,120]. According to the guidelines, “practical guidance from the Heart Failure Association of the European Society of Cardiology Study Group on peripartum cardiomyopathy”, it is crucial to assess the degree of cardiopulmonary distress in all patients. Patients with cardiopulmonary distress should be treated in a cardiac intensive care unit. These are patients who are hemodynamically unstable, who have respiratory distress syndrome, or signs of tissue hypoperfusion with abnormal oxygen metabolism in cells. Additionally, the assessment of distress must include clinical parameters such as the patient’s level of consciousness, mottled skin, and cold extremities, which may indicate shock, as well as the development of acute renal insufficiency [115]. The goal of treatment is to achieve hemodynamic stability, alleviate symptoms, prevent complications, and ensure fetal safety. As with other forms of HF, early initiation of treatment is of great importance as it significantly improves outcomes for these patients [121,122]. All patients must be hospitalized in intensive care units and have continuous non-invasive or invasive monitoring of vital functions.

Non-invasive hemodynamic monitoring provides physiological information while avoiding the risks of invasive monitoring. Invasive procedures involve puncturing the skin to insert a cannula or catheter into a blood vessel, cardiac chamber, or both, which can lead to the development of numerous complications [123]. Accordingly, it is necessary to regularly monitor systolic blood pressure, heart rate and rhythm, peripheral blood oxygen saturation using pulse oximetry, and urine output relative to the administered fluid volume. It is important to note that HF treatment guidelines do not recommend routine **oxygen administration** in patients without reduced oxygen saturation, as its use may lead to vasoconstriction and a reduction in cardiac output. A randomized study that followed the effects of oxygen therapy in HF patients showed that the reduction in cardiac output mediated by oxygen was mainly due to a decrease in heart rate, while the impact on stroke volume was minimal [124]. The harmful effect of hyperoxia is that there is an increase in reactive oxygen species, which reduces the bioavailability of nitric oxide in the blood vessels. Hyperoxia may also lead to a decrease in the synthesis of vasodilatory prostaglandins in the blood vessels [125,126]. Oxygen therapy should be provided to maintain arterial oxygen saturation at 95% or higher [114]. Severe respiratory failure during pregnancy can lead to increased fetal morbidity and mortality, with prolonged hypoxia associated with up to an 80% chance of preterm delivery and a 57% chance of early pregnancy loss [127,128]. Most patients with PPCM only require oxygen administration via nasal cannulas or oxygen masks [129]. If arterial blood saturation remains suboptimal despite oxygen support via a mask, the use of non-invasive positive pressure ventilation (NIV) is indicated. The positive effects of NIV in patients with cardiogenic pulmonary edema have been demonstrated [130,131,132]. In patients with HF, NIV reduces left ventricular pressure, thereby decreasing afterload in the left ventricle without reducing the cardiac index [133]. A study comparing the effectiveness of standard oxygen therapy, CPAP, and NIV in patients with cardiogenic pulmonary edema showed that the application of CPAP and NIV leads to faster regression of disease symptoms and improvement of metabolic status compared to conventional oxygen therapy. However, despite the proven effects of non-invasive mechanical ventilation on clinical parameters, no improvement in survival rates was observed between the two groups of patients [134]. Similar data were shown in a study by Plaisance P. et al. when, compared to usual medical care, the immediate application of CPAP alone in out-of-hospital treatment of acute cardiogenic pulmonary edema significantly improved physiological variables and symptoms and significantly reduced the incidence of tracheal intubation and in-hospital mortality [135]. The results of meta-analyses indicate the fact that the use of NIV can reduce respiratory distress in patients with acute left ventricular failure, reduce the need for intubation, and reduce mortality. NIV should not be considered a treatment option for patients who are non-compliant, have altered consciousness, or are experiencing respiratory or cardiac arrest [136]. NIV (non-invasive ventilation with a positive end-expiratory pressure of 5–7.5 cm H_2_O) has proven to be adequate respiratory support in pregnant women who had pneumonia, pulmonary edema in preeclampsia, ARDS, respiratory insufficiency due to myopathies, or spinal deformities [137,138,139,140,141]. For patients who, despite all forms of oxygen therapy, do not experience improved oxygenation—maintaining a state of hypoxia—or exhibit altered consciousness, intubation and connection to mechanical ventilation are necessary. 

Throughout pregnancy, physiological changes in the body can affect both the pharmacodynamics and pharmacokinetics of medications. However, specific dosing recommendations for obstetric patients during pregnancy are still lacking. In the third trimester of pregnancy, when PPCM is most prevalent, metabolic changes occur that are associated with modifications in the processes of both drug absorption and excretion [142,143]. Changes in the pharmacokinetics and pharmacodynamics of drugs should lead to adjustments in dosing regimens, which would result in better efficacy and reduced toxicity. **Diuretic therapy** is necessary for patients with pulmonary congestion and volume overload. Therapy should begin with loop diuretics. In the absence of signs of pulmonary congestion, diuretics should not be routinely administered to all patients with PPCM, as it has been shown that these medications can reduce placental blood flow and potentially affect the fetus [144]. The bioavailability of furosemide is approximately 60% in the general population. In the blood, nearly 99% of furosemide binds to albumin [145]. Studies have shown that furosemide is excreted in unchanged form via urine up to 70% and the excretion of the unchanged form of this drug depends on the organic anion transporter (OAT1) [146]. The remaining 30% is excreted in the form of glucuronide metabolites through the urine. This form depends on renal uridine diphosphate-glucuronosyltransferase (UGT) [146,147]. The study by Goncalves PVB et al. revealed that pregnant women have approximately 6 times greater volume of distribution and 4 times lower peak plasma concentration (Cmax) compared to established literature data. This increased volume of distribution during pregnancy may be attributed to a reduction in plasma proteins, to which furosemide is highly bound, as well as a dilution effect resulting from the increase in plasma volume [146]. According to the mentioned study, the increased clearance of furosemide could be attributed to increased induction of UGT during late pregnancy. Since albumin levels decrease and OAT expression increases during pregnancy, elimination is largely dependent on OAT1 and OAT3-mediated secretion [148]. It has been shown that furosemide crosses the uteroplacental barrier [149]. Furosemide’s passage through the placenta is incomplete, reaching approximately 40% [147]. According to leading consensus, an initial dose of 20–40 mg i.v. furosemide is recommended [7]. According to individual case reports, pregnant women treated for pulmonary edema with high doses of furosemide in the third trimester developed reversible oligohydramnios [118]. It has also been shown that the use of furosemide can stimulate urine production in the fetus [150]. Electrolyte disturbances in the mother due to furosemide use (hyponatremia/hypokalemia) can lead to electrolyte disturbances in the fetus, which can be symptomatic. In AHF, it is necessary to monitor fluid intake and excretion when using diuretics, as well as to regularly monitor renal function and electrolyte status. A study by Vigil-De Gracia P. et al. that examined the use of amlodipine, furosemide, and aspirin in pregnant patients did not show significant differences in preterm births, birth weight, or maternal and fetal complications [151]. According to the ROPAC study, the mean birth weight of newborns was lower in pregnant women who used diuretics compared to those without therapy [152]. In the aforementioned study, it was shown that the use of diuretics is associated with higher fetal mortality compared to the group of pregnant women who did not receive diuretics (4.3% versus 1.1%), and there were more frequent premature births in the group of patients who were treated with diuretics (31% vs. 11%) [152]. Today, the concurrent use of furosemide with other potentially ototoxic and nephrotoxic drugs is advised against.

**Intravenous nitrate** is recommended for normotensive patients with systolic blood pressure above 110 mmHg. The initial dose should be 10 µg/min, and it is advised not to exceed a maximum dose of 200 µg/min [7]. Glyceryl trinitrate (GTN) can cause uterine relaxation through processes mediated by nitric oxide and cyclic guanosine monophosphate (cGMP) [153]. The effect of GTN on the process of organogenesis has been studied in animals. Treatment with GTN caused various malformations in animal embryos and in vitro models [154,155]. There is limited data in the human population on the long-term use of GTN. According to the FDA, the use of GTN during pregnancy is classified as a Category B drug. At high doses, bradycardia was observed in the fetus, which did not reoccur after the dose was reduced [7]. Glyceryl trinitrate is the drug of choice in pregnant women with preeclampsia and acute left ventricular failure [7,156]. It is administered as an intravenous infusion starting from 5 µg/min up to a maximum dose of 100 µg/min [7,156]. Studies have shown that **sodium nitroprusside**, during administration, is metabolized into thiocyanate, which can cross the placenta and potentially cause cyanide toxicity in the fetus. Due to this risk and the possible reduction in blood flow through the placenta, its use during pregnancy is not recommended [157,158]. Patients who are hemodynamically unstable (in cardiogenic shock) and require **vasopressor and inotropic support** should be transferred as soon as possible to a clinical center that has the capability for mechanical circulatory support, ventricular assist devices (Impella, **left ventricular assist device**—LVAD), as well as heart transplant teams. The use of vasopressors and inotropes is necessary in all patients who develop hypotension or cardiogenic shock. Analysis of data from the German PPCM registry showed that all patients with PPCM treated with dobutamine required either heart transplantation or LVAD, while 95% of patients who did not receive dobutamine, despite similar initial LV function impairment, experienced cardiac function recovery without the need for support or heart transplantation [159]. According to their suggestions, a possible explanation for why dobutamine may exacerbate heart damage and HF progression could be the frequently observed low cardiac expression of signal transducer and activator of transcription 3 (STAT3) in patients with PPCM [159,160]. In the study by Labbene I. and colleagues, which examined the role of levosimendan in patients with PPCM and cardiogenic shock, rapid improvement in hemodynamics and LVEF was observed after treatment with the drug [161]. In contrast to adrenaline and dobutamine, levosimendan does not raise the heart’s need for oxygen. Levosimendan increases the sensitivity of troponin C to intracellular calcium, thereby extending the duration of the actin-myosin cross-bridge interaction [162]. By opening ATP-sensitive potassium channels in vascular smooth muscle cells, levosimendan leads to peripheral vasodilation, reduction in preload and afterload, and consequently further increases cardiac output [163]. The use of noradrenaline is also indicated in patients with cardiogenic shock. The potential harmful effects of inotropic agents and vasopressors on fetal development in humans remain uncertain. In women who remain in shock despite adequate medical therapy, **mechanical circulatory support** may be required, such as an intra-aortic balloon pump (IABP), percutaneous or durable ventricular assist devices (VAD), or extracorporeal membrane oxygenation (ECMO), as a bridge to recovery or transplantation [119]. If adequate oxygenation is needed for patients, early choices could include an intra-aortic balloon pump (IABP), Impella, or surgical methods (e.g., CentriMag, Abiomed BVS 5000). Impella has been demonstrated as an effective option for early improvement and recovery of cardiac function in women with severe PPCM and cardiogenic shock [164]. If weaning from mechanical circulatory support cannot be achieved within 7–10 days, it is essential to consider transitioning to a more durable mechanical circulatory support device (such as BiVAD, LVAD, or ECMO) [119]. Among patients with a severe form of PPCM who received an LVAD, 6% recovered and even 48% were indicated for a heart transplantation [165,166].

**Heart transplantation** is indicated for patients in whom methods of mechanical circulatory support cannot be implemented or recovery of the left ventricle has not been achieved after 6–12 months from the onset of the disease [119,167]. Heart transplantation should be delayed as long as possible because late recovery of cardiac function has been observed in PPCM patients.

Pregnancy itself represents a prothrombotic state, likely arising as a physiological response to reduce the risk of bleeding during the antenatal period, especially postpartum. Epidemiological data suggest that hypercoagulability begins early in pregnancy, with an increased risk of thrombosis starting from the first trimester. During pregnancy and the postpartum period, the risk of thromboembolic complications increases up to fivefold compared to the non-pregnant state [168]. An increase in the level of coagulation factors such as VII, VIII, X, and von Willebrand factor is observed during normal pregnancy while factors II, V, and IX remain unchanged. Additionally, fibrinogen concentrations can rise significantly, reaching up to three times their usual levels [169,170,171]. During a normal pregnancy, there is a significant decrease in the level of free protein S, especially at the beginning of pregnancy, while in the last trimester, the levels normalize [172]. Throughout pregnancy, levels of plasminogen activator inhibitor type 1 are increased as much as fivefold.

In addition to pregnancy itself being a procoagulant state, when combined with HF and reduced LVEF, the risk of thrombotic complications significantly increases, making **anticoagulant therapy** necessary. To prevent systemic thromboembolism, anticoagulation is recommended for women with PPCM and EF < 35%, according to the European Society of Cardiology, or in patients with PPCM and EF < 30%, according to the American Heart Association [7,30,119]. Numerous cases of intraventricular thrombosis have been described in patients with PPCM [173,174,175]. Kane A. and colleagues showed in their study that even 30% of PPCM patients had intraventricular thrombosis proven by transthoracic and transesophageal echocardiography [176].

Anticoagulant drugs used during pregnancy include low molecular weight heparin (LMWH) or unfractionated heparin (UFH), and after childbirth, you can switch to warfarin. UFH, LMWH, and danaparoid sodium do not cross the placenta [177]. A study that examined the use of UFH in pregnant PPCM patients found that the rates of congenital abnormalities, stillbirths, or preterm births are approximately equivalent to those in the general population [178]. When there is a risk of bleeding during pregnancy, UFH is safer due to its short elimination half-life and the availability of an antidote—protamine sulfate [179,180,181]. On the other hand, UFH is more negatively associated with the occurrence of heparin-induced thrombocytopenia and osteoporosis [182,183]. Data from the literature and clinical experience support the safe use of LMWH during pregnancy [184,185].

It has been shown that coumarin-based medications cross the uteroplacental barrier and have both teratogenic effects and other potentially harmful effects on the fetus, such as bleeding [186]. Warfarin is reserved only for pregnant women with mechanical artificial valves [30,186]. The most common occurrence of embryopathies is observed during the early stages of pregnancy (first trimester), with the risk to the fetus decreasing over time [186,187,188]. The teratogenic effects seem to be dose-dependent, with the highest margin of safety associated with doses below 5 mg/day [188,189,190]. Experts have recommended low-dose warfarin (less than 5 mg daily) throughout pregnancy in women with mechanical valves, given a very low frequency of fetal anomalies [189,190]. According to a Chinese study by Li T and colleagues, fetal complications occurred across all trimesters (first, second, and third trimesters: 19.2%, 9.9%, and 8.0%, respectively) in pregnant women taking warfarin [191]. According to their study, pregnant women who took warfarin daily doses of ≤5 mg might have only around a 60% chance of giving birth to a live baby without maternal complications. According to current guidelines, the risk to the infant from maternal warfarin use during breastfeeding is considered low. Even high doses of 25 mg are deemed safe for use during lactation [192]. The use of LMVH during lactation is safe [192]. Although pregnant women are excluded from studies involving new oral anticoagulants (NOACs), there are individual cases of their use during pregnancy [193,194]. The limited available evidence raises concerns regarding embryonic-fetal safety, with a high incidence of miscarriage and a 4% rate of anomalies associated with rivaroxaban use [195]. The Royal College of Obstetricians and Gynaecologists guidelines do not support the use of NOACs during breastfeeding [196]. The consensus from the European Society of Cardiology also does not recommend the use of NOACs during pregnancy [197]. Some studies have indicated that the use of bromocriptine carries an increased risk of thromboembolic complications in patients with PPCM [198,199]. The ESC expert consensus recommends anticoagulation with heparin to avoid cardioembolic complications in patients treated with bromocriptine (if no contraindication exists) [7,119]. When it comes to deciding on the use of prophylactic or therapeutic doses of anticoagulant therapy in patients with PPCM, there are no clear guidelines. It is best to rely on clinical experience with continuous reevaluation of the thrombotic and hemorrhagic risk for each patient individually.

**Bromocriptine** is classified as a dopamine D2 agonist and is an FDA-approved drug indicated for disorders that cause hyperprolactinemia. By suppressing prolactin synthesis, bromocriptine leads to the cessation of lactation. Numerous case reports show that the use of bromocriptine has positive effects in patients with PPCM [200,201,202]. Haghikia A. and colleagues studied the effects of bromocriptine on the outcomes of patients with PPCM and impaired right ventricular function [203]. Right ventricular dysfunction has been identified as a poor prognostic indicator in patients with PPCM. Despite its association with adverse outcomes, evidence suggests that bromocriptine administration in patients with PPCM and impaired right ventricular function may lead to substantial recovery. Long-term follow-up of PPCM patients in Canada (follow-up period 20 years) confirmed that patients who receive bromocriptine for 6 months have a better recovery of LVEF compared to those who do not receive this specific treatment. Also, the long-term improvement of LV function was better in patients treated with bromocriptine [204]. A recent meta-analysis of eight studies (two randomized-controlled, six observational) showed that the use of bromocriptine in patients with PPCM leads to higher survival [205]. A meta-analysis by Kumar A. and colleagues also confirmed the above and showed that the use of bromocriptine in women with PPCM reduces the degree of LVEF dysfunction and reduces mortality in this population [206]. The ESC guidelines recommend the use of bromocriptine for patients with PPCM (Class IIB, Level of Evidence B). The dosage of bromocriptine for treating PPCM may depend on the severity of the condition. For patients with milder clinical presentations, a daily dose of 2.5 mg for 7 days is generally sufficient. Conversely, for patients with severely reduced EF in cardiogenic shock or those with predictors of poor outcomes, higher doses may be administered over a 6-week period (2.5 mg twice daily for the first 2 weeks, followed by 2.5 mg once daily for the subsequent 6 weeks) [119]. As some studies have shown increased prolactin release during ECMO, bromocriptine is advised at a dose of 10 mg twice daily, which is also the maximum dose of this medication [115].

### 7.2. Therapy for Stable Patients

Treatment and medical monitoring from the very beginning of the disease is the most important in hemodynamically stable patients with PPCM. It is also crucial to consider whether the disease developed during pregnancy (and at which gestational week) or after childbirth. During pregnancy, it is important to consider the possible adverse effects of medications on the fetus. After childbirth, if breastfeeding is not stopped, the concentration of the drug in the mother’s milk and the side effects on the infant must be taken into account.

The use of **beta-blockers** is indicated for all patients with PPCM who do not show signs of pulmonary congestion. Beta-blockers can affect the tone of the uterus, so only β1-selective blockers are used before childbirth [141]. Metoprolol succinate (a β1-selective blocker) is favored to avoid interfering with β2-mediated uterine relaxation and peripheral vasodilation [207]. Administration of metoprolol may be associated with slowed heart rate and hypoglycemia in the fetus [208]. According to a study by Tanaka K. and colleagues, the use of beta-blockers was associated with a reduction in the birth weight of newborns [209]. Among the beta blockers, carvedilol is a non-selective (alpha and beta) blocker that has been shown not to cause fetal growth disorders [209]. If beta-blockers are used before delivery, newborns must be observed for 24–48 h due to the potential for hypotension, bradycardia, or hypoglycemia. The study by Duan L. and colleagues did not show cardiovascular malformations with the use of beta-blockers [210]. Additionally, the study by van de Vusse D. did not show teratogenic effects with the use of labetalol during pregnancy [211]. Lipophilic drugs such as labetalol easily cross the uteroplacental barrier [212]. Despite its significant placental transfer, labetalol is considered a safe beta-blocker for use during pregnancy. Bisoprolol, carvedilol, and metoprolol succinate, which are classified as Category C by the FDA, can be used during pregnancy and breastfeeding. When it comes to the choice of diuretics, hydrochlorothiazide is the drug of choice. Thiazide diuretics lead to potassium loss and reduced plasma volume, and they also inhibit the excretion of uric acid. Hydrochlorothiazide crosses the placenta [213]. According to the database, the risk of gross structural malformations with the use of hydrochlorothiazide was 4.2%, slightly higher than expected, but there was no increase in specific malformations. The use of diuretics in patients with PPCM can reduce blood flow to the placenta [150]. Thiazide diuretics can cause hyponatremia, hypokalemia, and hyperglycemia in both pregnant women and fetuses. Their effects on smooth muscles may inhibit contractions and delay labor. According to the FDA, it falls into Category B.

The use of nitrates and digoxin is considered safe during pregnancy [7,197]. ACE inhibitors and angiotensin receptor blockers (ARBs) (Category D) are contraindicated in pregnancy and should be discontinued before conception due to serious fetotoxicity [214,215]. Damage to fetal kidney function, oligohydramnios, anhydramnios, bone defects, contractures, and fatal outcomes have been reported in mothers using ACE inhibitors during the later stages of pregnancy (second and third trimesters) [115,216,217]. In a series of cases involving pregnant women exposed to ARBs during late pregnancy, fetotoxicity associated with their use has been demonstrated [218]. The critical period for symptoms of fetopathy begins at the 20th week of pregnancy.

**Fetopathy** is clinically characterized by oligohydramnios or anhydramnios and occurs due to fetal kidney dysfunction, which can be temporary and mild but also severe and irreversible [217,219]. A study by Cooper WO and colleagues found that the use of ACE inhibitors was associated with an increased risk of cardiovascular and central nervous system malformations (OR, 3.72; 95% CI, 1.89 to 7.30) and (OR, 4.39; 95% CI, 1.37 to 14.02), respectively [220]. In patients using ACE inhibitors, it is necessary to discontinue them and replace them with other antihypertensive agents as soon as possible when pregnancy begins, if possible, during the first trimester due to the more frequent occurrence of fetal damage later in pregnancy. In addition to ACE inhibitors, **angiotensin receptor neprilysin inhibitors** are also contraindicated for treating heart failure during pregnancy. **Mineralocorticoid receptor inhibitors** belong to Category D and are contraindicated during pregnancy. Spironolactone shows a significant anti-androgenic effect and crosses the placenta, which can affect fetal development [207]. Data for eplerenone are unavailable, but it is considered contraindicated. The use of ivabradine is considered contraindicated during pregnancy. According to a series of cases of pregnant women exposed to ivabradine, it appears not to be a major teratogen [221]. However, earlier studies conducted on mice showed its harmful effects.

Data from the literature indicate that the use of sodium-glucose co-transporter-2 (**SGLT2**) inhibitors in animal models (rats and rabbits) may have adverse effects, including increased risk of major congenital anomalies, decreased fetal weight, and delayed bone maturation. In addition, adverse effects have been reported in newborn children of mothers who used SGLT2 during pregnancy [222]. Additionally, there is evidence of the harmful effects of **GLP-1** agonists and **SGLT2 inhibitors** [223].

After delivery, it is essential to determine whether the woman is breastfeeding when considering the choice of heart failure medication. For women who continue breastfeeding, it is important to consider whether the medications pass into breast milk and their concentration in the milk due to their potential harmful effects on the newborn. The use of ACE inhibitors is justified during breastfeeding with caution [119]. Limited information indicates that only small levels of perindopril are found in breast milk, consistent with other drugs in this class [224]. Similar data exist for the use of enalapril and captopril during breastfeeding [225,226,227]. Administration of enalapril and captopril during breastfeeding is associated with small amounts of these drugs in milk, so no side effects are expected in breastfed babies [227]. There is no available information on the use of lisinopril and fosinopril during breastfeeding, so preference should be given to well-studied drugs. However, sacubitril/valsartan (Entresto), despite its effectiveness, is not recommended during lactation due to possible adverse effects on the newborn [228]. Results of the study of Falconia S. and colleagues are contradictory and indicate that sacubitril/valsartan is excreted in milk in very low concentrations and that the risk of adverse effects on the newborn is very low [228]. In women who are not breastfeeding or whose lactation has been discontinued after delivery and have developed PPCM, they should be treated according to current heart failure guidelines without restrictions in medication use, with regular monitoring by a gynecologist and cardiologist. When it comes to the duration of medication for treating heart failure, data are inconsistent. The use of medications is recommended for at least 12–24 months after full recovery of LV function. Some authors propose lifelong use of medications even after complete recovery of heart function, while others advise gradual discontinuation of treatment.

## 8. Delivery of Patients with PPCM

The method of delivery (cesarean section or natural vaginal delivery) as well as the timing of delivery is determined by the hemodynamic status of the patient, as well as obstetric and gynecological indications. The highest priority is the survival of the mother, but the condition of the fetus can also dictate the initiation of delivery. Patients with hemodynamic instability despite treatment should undergo emergency delivery regardless of gestational age [229]. A cesarean section is recommended when the patient is hemodynamically unstable, on inotropic support or mechanical circulatory assistance, along with combined spinal and epidural analgesia. It is important that this decision is made by a multidisciplinary team. Patients with adequate cardiac output may tolerate induction and vaginal delivery.

## 9. Prognosis

Long-term outcomes vary. Approximately 25% of patients develop HF (from mild to severe form), and the remaining patients die during the course of the disease. It is advised that patients with PPCM do not plan a new pregnancy if the LVEF is low. If, despite the advice, the woman’s desire for offspring is strong, the patient should wait at least 5 years after the normalization of LVEF. Certainly, every new pregnancy carries a high risk for a woman with a previous history of PPCM [230].

## 10. Conclusions

Peripartum cardiomyopathy is a significant cause of maternal morbidity and mortality worldwide. The importance of this condition is immense because it often threatens two lives—the life of the mother and the life of the fetus. The risk factors that may lead to PPCM are not yet fully understood. Various socioeconomic or genetic factors may play a significant role in the development of PPCM, although their prevalence and significance are still debatable. The varying incidence of this condition in different regions of the world is due to inadequate diagnosis of the disease, given the often poorly defined criteria and the absence of pathognomonic tests. When it comes to treating this disease, many dilemmas remain regarding medication dosage regimens and the safety of their use during pregnancy and lactation. We particularly emphasize the importance of organizing databases on the use of medications during pregnancy and any potential adverse effects of these medications on both the mother and the fetus. In terms of the challenges faced by patients with PPCM, most authors still rely on data from older, smaller studies or case reports, making multicenter studies with larger patient cohorts necessary in the future to provide more relevant data. To avoid having the future mirror the present, additional research is needed to explore new drugs used in the treatment of heart failure, and which are not currently part of the therapeutic regimen in patients with peripartum cardiomyopathy. PPCM is a disease that we must think about, and where there are still therapeutic challenges.

## Data Availability

Not applicable.

## Supplementary Materials

The following supporting information can be downloaded at: https://www.mdpi.com/article/10.3390/ijms251910559/s1.
