# Supplementary figures and images for: What Do We Know about Peripartum Cardiomyopathy? Yesterday, Today, Tomorrow
Source: Int J Mol Sci. 2024 Sep 30;25(19):10559. doi: 10.3390/ijms251910559 (PMC11477285; doi:10.3390/ijms251910559)

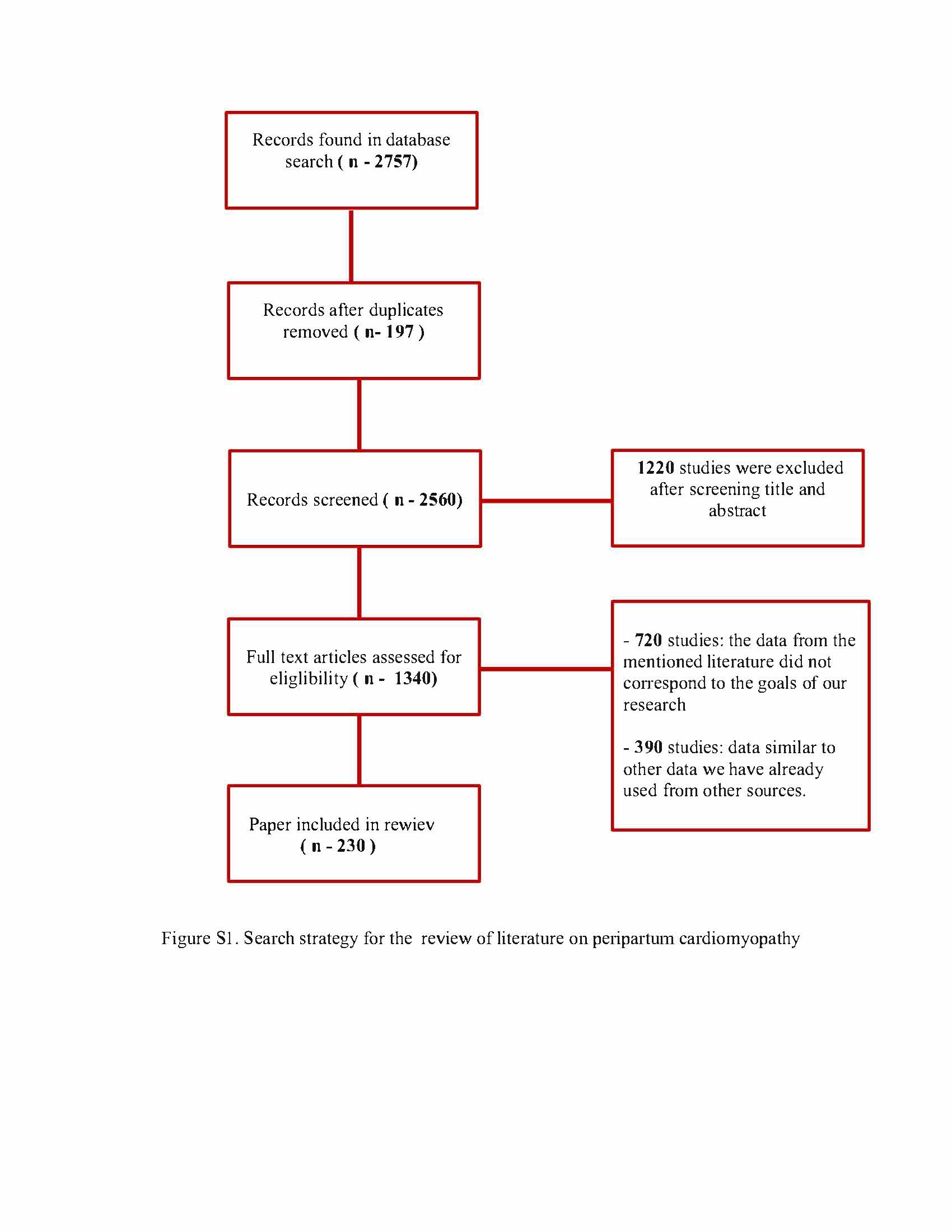

Supplement: Supplementary file 1 [file ijms-25-10559-s001.zip › ijms-3219280-supplementary.jpg]
